# Impact of long working hours on depressive symptoms among COVID-19 frontline medical staff: The mediation of job burnout and the moderation of family and organizational support

**DOI:** 10.3389/fpsyg.2023.1084329

**Published:** 2023-02-15

**Authors:** Chengjie Yin, Jingya Ji, Xin Cao, Hui Jin, Qiang Ma, Yuexia Gao

**Affiliations:** ^1^Department of Health Management, School of Public Health, Nantong University, Nantong, Jiangsu, China; ^2^Department of Hospital Management, Affiliated Hospital of Nantong University, Nantong, China

**Keywords:** working hours, job burnout, family support, organizational support, depression, COVID-19 frontline medical staff

## Abstract

**Background:**

During the COVID-19 pandemic, the frontline medical staff faced more workload and heavier physical and mental stress, which increased their job burnout and negative emotions. However, little is known about the potential factors mediating and moderating these relations. This study investigates the association between long working hours and depressive symptoms among frontline medical staff in China, and explores the potential mediating effect of job burnout, and moderating effect of family and organizational support on these associations.

**Methods:**

Data of 992 frontline medical staff who participated in the prevention and control of COVID-19 was obtained from the online survey conducted in November to December 2021 in China. Depressive symptoms were evaluated using the Patient Health Questionaire-9 (PHQ-9). Moderated mediating model was employed to understand the relationship between long working hours (X), depressive symptoms (Y) mediated through job burnout (M), moderated by family support (W1) and organizational support (W2), while controlling all possible covariates.

**Results:**

56.96% of participants worked more than 8 h per day. 49.8% of them had depressive symptoms (PHQ-9 ≥ 5) and 65.8% experienced job-related burnout. Long working hours was positively associated with depressive symptoms score (*β* = 0.26, 95% CI:0.13 ~ 0.40). Mediation analyses revealed that job burnout significantly mediated this relationship (indirect effect = 0.17, 95% CI: 0.08 ~ 0.26). Moderated mediation further indicated that both two interactions of social support (family support W1, organizational support W2) and job burnout were negatively related to depressive symptoms among frontline medical staff, indicating that higher social support being less job burnout with lower depressive symptoms.

**Conclusion:**

Longer working hours and higher job burnout may contribute to worse mental health among frontline medical staff. Social support could buffer the detrimental effects by reducing their job burnout.

**Contribution:**

The main contribution of this study was to estimate the negative effect of long working hours on depressive symptoms among frontline medical staff and explore the potential mediating role of job burnout and moderating role of social support on these associations.

## 1. Introduction

The World Health Organization declared coronavirus disease 2019 (COVID-19), which began in Wuhan City in December 2019 and rapidly spread worldwide, as an international public health emergency (Williams et al., 2020). The pandemic is characterized by fast transmission, a wide range of infections, great difficulty in prevention and control and infection to medical staff, posing great challenges and pressure to the existing public health emergency response system (Allsopp et al., 2019). During the COVID-19 pandemic, frontline medical staff experienced longer working hours, more heavier workload and higher work-family imbalance (Walton et al., 2020), resulting to their burnout, which in turn affect their physical and mental health. The costs of job burnout were extremely high due to its direct impact on employee’s health, low productivity, absenteeism, and turnover. Therefore, it is very crucial to understand whether and how work-related demands (long working hours) impact depressive symptoms among frontline medical staff and explore the potential moderating role of social support (family support and organizational support) in these associations. Few studies focused on the possible links between long working hours and mental health status among frontline medical staff are available. Social support may buffer the detrimental effects of long working hours and burnout on mental health in medical staff during the COVID-19 pandemic. Therefore, we aimed to explore the potential factors impact on their mental health and moderating effect of social support on the association between long working hours and depression.

### 1.1. Long working hours and depressive symptoms

The job-demand-resource (JD-R) theory (Demerouti et al., 2001) has been widely adopted to investigate the association between job demands that function as stressors and are hazardous to health and the resources that counter for the stress effects of job demands. Job demand are defined as those physical, psychological, social, or organizational aspects of the job that require sustained effort, which create to job burnout, absenteeism and depression (Bakker and Demerouti, 2017). During the COVID-19 pandemic, frontline medical staff faced various stresses, such as increasing work overload, long working hours, blurring work-life boundaries, patients demands and physical demands, all of which could lead to their burnout and health impairment (Kang et al., 2020). Previous studies revealed that the medical staff were at a higher risk of suffering from mental health problems during severe acute respiratory syndrome, H1N1, and Ebola pandemics (Tam et al., 2004; Goulia et al., 2010; Ji et al., 2017). Given the scope of the pandemic, COVID-19 is expected to have a more significant psychological impact than earlier pandemics (Rossi et al., 2020; Tan et al., 2020). COVID-19 pandemic triggered negative emotions, which is more common among frontline medical staff (Sanghera et al., 2020; Greene et al., 2021). Existing studies showed that the incidence of depression or depressive symptoms among medical staff (22.8–50.4%) (Lai et al., 2020; Pappa et al., 2020) was higher than the general population (18.25–23.33%) (Liang et al., 2020) during the COVID-19 pandemic. Studies have found that long working hours were associated with depression and anxiety (Bannai and Tamakoshi, 2014; Lang et al., 2020). A prospective cohort study of 2,123 British civil servants the odds ratio for a subsequent major depressive episode was 2.43 times higher for those working at least 11 h a day compared to individuals working for standard 7–8 h (Virtanen et al., 2012). However, little is known whether long working hours are related to depressive symptoms among frontline medical staff. Hence, we proposed that long working hours was positively associated with depressive symptoms (Hypothesis 1).

### 1.2. The mediating role of job burnout

Job burnout is a job-related psychological symptoms that derived from prolonged exposure to chronic stressors, eventually leading to a physical, emotional and mental status of feeling drained (Maslach et al., 2001). Continuous accumulation of work pressure and the declination of job satisfaction among employees engaged in the service industry, resulting to individual burnout in various aspects of emotion and behavior (Bakker and de Vries, 2021). Medical staff are one of the high-risk groups of job burnout worldwide (Hyman et al., 2011). A recent cross-sectional study of 15,627 participants from 459 township hospitals in six provinces of China showed that 47.6% of the respondents experienced moderate job burnout, and 3.3% experienced severe job burnout (Xu et al., 2020). The determinants and effect of job burnout were widely reported in existing research. A systematic review of 768 studies about nursers’ stress and burnout found that high level stress, coping mechanisms and job satisfaction were associated with the incidence of burnout symptoms (Friganović et al., 2019). Another recent meta-analysis from 67 studies with 84,169 participants also verified that job burnout were positively related to depression (Koutsimani et al., 2019). Whereas few studies examined the mediating role of job burnout among the linkage between long working hours and mental health among frontline medical staff, especially during the COVID-19 pandemic. According to the JD-R model and aforementioned studies, job-related burnout resulting from high work demands (e.g., longer working hours, heavier workload) had negative impact on job performance and individuals’ physical and mental health. Thus, we hypothesized that job burnout may mediated the association between long working hours and depressive symptoms (Hypothesis 2).

### 1.3. The moderating role of family and organizational support

Social support is a key construct in understanding depressive symptoms in the context of job demands. Many studies have theoretically and empirically demonstrated social support increased individual’s psychological resources and protected people from the adverse effects to life stress (Ganster and Victor, 1988). Family support and organizational support are vital components of social support. Family support from spouse and family members can provide emotional support, helping individuals relax their tensions, relieve work fatigue, and improve their work abilities (Langford et al., 1997). In Eisenberger’s theory of organizational support (1986), organizational support is defined as “the extent to which employees perceive that the organization values their contributions and cares about their well-being” (Rhoades and Eisenberger, 2002). The main effect model of social support (Langford et al., 1997) showed that individuals’ positive life experience and stable social network could promote their mental health. Many studies have demonstrated the protective effect of social support on healthcare workers during pandemics (Maunder et al., 2003; Shen et al., 2022). The stress-buffering model of social support indicates that perceived social support may help individuals coping stress events more positively, reducing negative emotions (HermanStahl and Petersen, 1996). A cross-sectional study using a nationwide sample of 11,419 Chinese science and technology professionals with various organizations have revealed that both work-related support and family support were negatively related to work-family interference, which in turn decreased their depressive symptoms (Gan et al., 2015). During the COVID-19 pandemic, the protective effect of organizational support on the work stress and mental health of frontline medical staff has also been verified (Zhou et al., 2021). Few studies indicated the multiple roles of family support in the effect of decreasing high job-related demands on depressive symptoms among frontline medical staff. Thus, it is essential to further explore the underlying mechanisms how social support attenuate the negative effect of job-related demands on mental health among medical staff during the COVID-19 pandemic. Therefore, based on social support model, we proposed the following hypothesis that social support can buffer the direct and indirect relations between long working hours and depressive symptoms through job burnout (Hypothesis 3).

### 1.4. The present study

**Figure 1:**
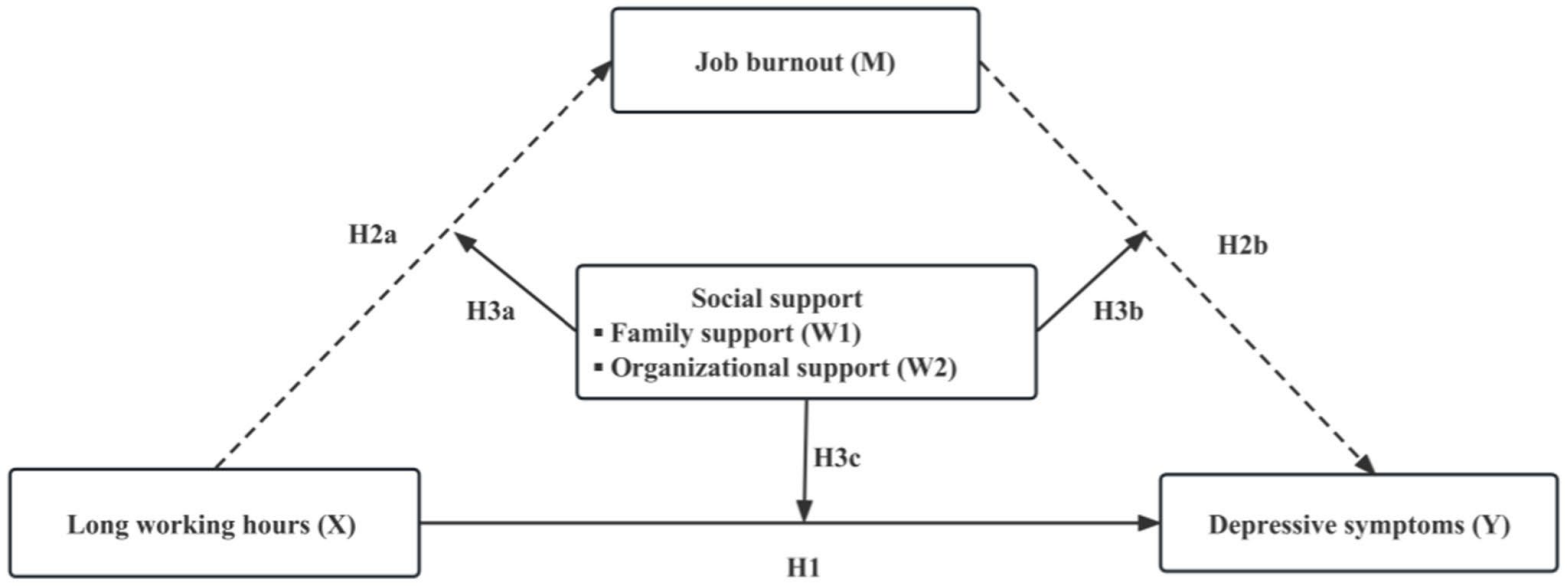
A hypothetical model of relationships among long working hours, job burnout, social support and depressive symptoms.

In this study, we examined a moderated mediation model of the association model of the association between long working hours, job burnout, social support (family support and organizational support) and depressive symptoms among frontline medical staff. The aims of this study were threefold: firstly we tested whether long working hours had positive impact on depressive symptoms among frontline medical staff. Secondly, we used mediation model to explore the mediating role of job burnout in the relationship between long working hours and depressive symptoms. Thirdly, we test the moderating role of social support in the direct and indirect relations among them. Take together, the proposed model is presented in Figure 1.

## 2. Materials and methods

### 2.1. Study design and participants

This cross-sectional survey was conducted in the Jiangsu province in eastern China in collaboration with local health bureaus from November and December 2021. Participants were frontline medical staff worked in medical units such as disease control and prevention (CDC), hospitals, primary health centers and other medical units during the COVID-19 pandemic prevention and control. Data were collected by online survey and 1,200 medical staff completed the questionnaires. In total, 992 valid questionnaires were recovered, with response rate being 82.67% (992 out of 1,200). Cases with missing data in the surveys (126 cases without working hours per day) or with wrong information (82 cases do not work in frontline COVID-19 pandemic) were excluded. All participants provided their online written informed consent in the study. This study involving human participants was reviewed and approved by the Ethics Committee of Nantong University.

### 2.2. Measures

#### 2.2.1. Predictor variable

Working hours was measured using the following question “How many hours did you usually work per day on pandemic prevention and control in the last month?” In the present study, working hours was categorized into ≤8, >8 h per day reflecting working conditions of frontline medical staff. As for the categorization, the China Labor Standards Act sets 8 h per day as legal working hours or 44 h per week as legal working time.

#### 2.2.2. Mediating variable

Job burnout was evaluated using the General Version of the Burnout Scale (MBI-GS; Goldberg et al., 1996). The 15 items scale measured three dimensions of job burnout, with 5 items for emotional exhaustion, 4 items for depersonalization, and 6 items for low personal accomplishment. Each items of the scale scored using a 6-point Likert scale ranging from 0 (“never”) to 6 (“every day”). A high score for emotional exhaustion and depersonalization dimensions indicated more severe burnout. For low personal accomplishment, a lower score indicates more severe burnout. The comprehensive burnout score was calculated using the following formula: 0.4 * emotional exhaustion +0.3 *depersonalization +0.3 * (reduced personal accomplishment)/15. Cut-off points adopted were used to classify into three groups based on their mean score (Golonka et al., 2019), no burnout (0–1.49), mild to moderate burnout (1.5–3.49), severe burnout (3.5–6.0). The Cronbach’s alpha of MBI-GS in this study was 0.904.

#### 2.2.3. Moderating variables

Family support was measured by a single item “How much do your family members support you work on pandemic prevention and control?” The item was scored on a five-point Likert scale, range from l (very unsupported) to 5 (very supported), with high scores indicating greater family support.

Organizational support was assessed using 8-items scale of the perceived organizational support scale (Shen and Benson, 2016). Each items of the scale were rated on a 7-point Likert scale ranging from 1 (very disagree) to 7 (very agree). The final score is the sum of these 8 items, with higher scores indicating greater organizational support. Cronbach’s alpha of this scale in present study was 0.863.

#### 2.2.4. Dependent variable

Depressive symptoms was measured using the 9-item Patient Health Questionnaires (PHQ-9) (Kroenke et al., 2001), which is one of most widely used for scales for measuring mental disorder (Abe et al., 2020). Each item measures the frequency of depressive symptoms with a four-point Likert scale ranging from 0 to 3 (0 = not at all, 1 = several days; 2 = more than half the days, 3 = almost every day). The sum-score of nine items was categorized as four groups, non-depression (0–4), mild depression (5–9), moderate depression (10–14), and major depression (≥15) (Lu et al., 2018). Participants with sum-score more than 5 was considered to have depressive symptoms (Lin et al., 2021). Cronbach’s alpha of this scale in present study was 0.935, indicating a good internal consistency reliability.

#### 2.2.5. Control variables

The basic characteristics were included as covariates to control the effects as confounding factors: gender, age, marital status, education level, and work units. Ages were categorized into four groups (“<30,” “30–39,” “40–49,” and “≥50 years”).

### 2.3. Statistical analyses

Statistical analyses were conducted using SPSS 25.0 and Hayes (2015) SPSS macro program PROCESS. First, the basic characteristics of participants were described using summary statistics, that is, mean, standard deviations (continuous variables), frequency distribution and percentage (categorical variables). Bivariate analysis was calculated to examine correlations between the independent variables (X), mediator (M), moderator (W1, W2) and dependent variable depressive symptoms (Y). Second, the model 4 of the PROCESS macro was used to test the relationship between the long working hours (X) and depressive symptoms (Y) and the mediating role of job burnout (M) among this relationship. Model 1 examined the relationship between the long working hours (X) and depressive symptoms (Y) (H1 in Figure 1). Model 2 tested the relationship between the long working hours (X) and job burnout (M) (H2a in Figure 1) and model 3 tested the direct effect of the long working hours (X), job burnout (M) on depressive symptoms (Y) (H2b in Figure 1). This two models provided direct and indirect paths among these variables. The details for model 4 have been presented in Table 1.

**Table 1:** Mediation models of long working hours on depressive symptoms through burnout.

	Model 1 (Depressive symptoms)			Model 2 (Job burnout)			Model 3 (Depressive symptoms)		
	*β*		95% CI	*β*	*t*	95% CI	*β*	*t*	95% CI
Control variables									
Gender	−0.11	−0.23	−1.03, 0.81	−0.14	−1.83	−0.30, 0.01	0.45	1.27	−0.24, 1.15
Age	−0.02	−0.61	−0.07, 0.04	−0.01^**^	−2.69	−0.02, –0.00	0.03	1.52	−0.01, 0.07
Marital status	0.18	0.28	−1.05, 1.41	−0.05	−0.47	−0.26, 0.16	0.37	0.78	−0.56, 1.30
Education	0.80	1.84	−0.05, 1.65	0.05	0.72	−0.09, 0.19	0.59	1.81	−0.05, 1.24
Working units	−0.09	−0.43	−0.50, 0.32	−0.01	−0.20	−0.08, 0.06	−0.06	−0.39	−0.37, 0.25
Independent variable									
Working hours	1.83^***^	4.25	0.99, 2.68	0.14^*^	3.54	0.11, 0.40	0.83^*^	2.54	0.19, 1.47
Mediator									
Job burnout							3.92^***^	27.18	3.64, 4.21
Adj-*R*^2^	0.02			0.03			0.44		
*F*	3.64^**^			4.21^***^			111.03^***^		

*N* = 992; Gender: male = 0, female = 1; Marital status: Unmarried = 1, Married = 2, Others = 3; Education: Junior college = 1, Bachelor’s degree = 2, Master’s degree = 3; Working units: CDC = 1, Hospital = 2, Primary health centers = 3, Others = 4; Working hours: ≤8 h = 0, >8 h = 1. 95% CI = Bootstrap confidence intervals with lower and upper limits. *p* < 0.05, ^**^p < 0.01, ^***^p < 0.001.

**Figure 2:**
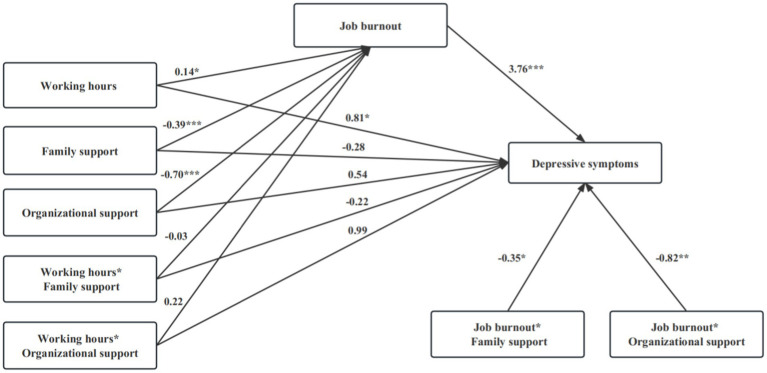
Results for the testing hypothesis. *p < 0.05, **p < 0.01, ***p < 0.001. Covariates: gender, age, marital status, education, and working units.

**Figure 3:**
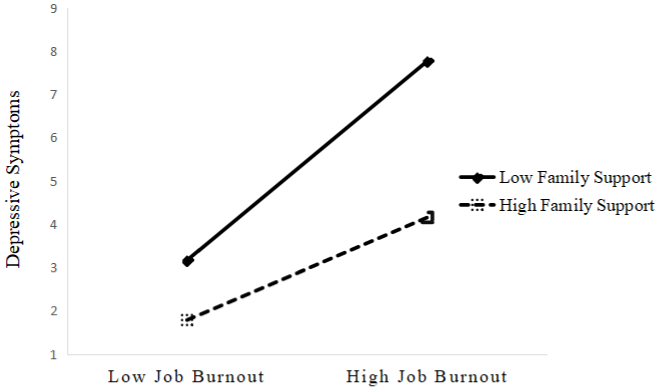
The moderating effect of family support on the relationship between job burnout and depressive symptoms.

**Figure 4:**
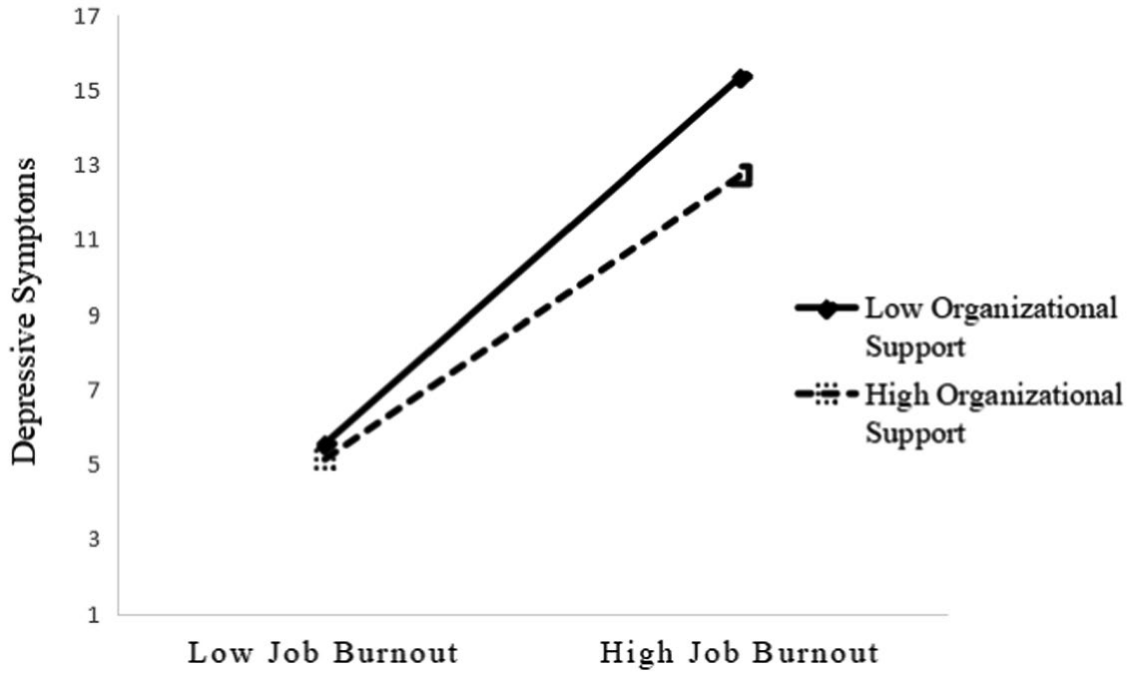
The moderating effect of organizational support on the relationship between job burnout and depressive symptoms.

Third, the proposed moderator variables of social support (W1, W2) were integrated into the model for testing the moderated mediation hypothesis (Hypothesis 3) using model 76 of the PROCESS macro. The moderating effect of social support (W1, W2) in the relation of long working hours to job burnout would be examined (H3a in Figure 1) in Model 4a. The moderating effect of social support (W1, W2) on both the partial effect of frontline medical staff job burnout on their depressive symptoms (H3b in Figure 1) and the direct effect of working hours on depressive symptoms (H3c in Figure 1) would be estimated in Model 4b. If the coefficients of the interaction of social support (W1, W2) and working hours and the interaction of social support (W1, W2) and job burnout were significant, indicating social support buffered the frontline medical staff from the negative effect by reducing their job burnout or depressive symptoms. The details for model 76 have been presented in Table 2 and Figure 2, the value of moderated indirect effects were calculated and presented in Table 3. The simple slope tests were conducted to describe the moderating effect of social support in Figures 3, 4. All the control variables were entered in all analysis. Both the mediation and moderated mediation analysis was performed based on 5,000 bootstrapped samples and bias-corrected 95% confidence intervals (CI) were calculated. If the confidence interval values do not contain zero, the mediating and moderated mediating effects can be considered significant.

**Table 2:** Moderated mediation regressions of long working, burnout, social support, and depressive symptoms.

	Model 4a (Job burnout)			Model 4b (Depressive symptoms)		
	*β*	*t*	95% CI	*β*	*t*	95% CI
Control variables						
Gender	−0.20^**^	−2.91	−0.33, –0.06	0.50	1.40	−0.20, 1.19
Age	−0.01	−1.77	−0.01, 0.00	0.04	1.73	0.00, 0.08
Marital status	−0.15	−1.70	−0.33, 0.02	0.40	0.85	−0.52, 1.33
Education	0.05	0.77	−0.07, 0.17	0.47	1.45	−0.17, 1.11
Working units	−0.01	−0.48	−0.07, 0.04	0.07	−0.45	−0.38, 0.24
Independent variable						
Working hours	0.14^*^	2.22	0.02, 0.26	0.81^*^	2.48	0.17, 1.45
Mediator						
Job burnout				3.76^***^	21.99	3.42, 4.09
Moderator						
Family support	−0.39^***^	−12.01	−0.46, –0.33	−0.28	−1.53	−0.64, 0.08
Organizational support	−0.70^***^	−10.96	−0.82, –0.57	0.54	1.53	−0.15, 1.24
Interactions						
Working hours* Family support	−0.03	−0.40	−0.16, 0.10	−0.22	−0.63	−0.92, 0.47
Working hours* Organizational support	0.22	1.69	0.03, 0.47	0.99	1.48	−0.33, 2.30
Job burnout* Family support				−0.35^*^	−2.39	−0.64, –0.06
Job burnout* Organizational support				−0.82^**^	−2.93	−1.37, –0.27
Adj-*R*^2^	0.29			0.46		
*F*	40.42^***^			63.56^***^		

*N* = 992; Gender: male = 0, female = 1; Marital status: Unmarried = 1, Married = 2, Others = 3; Education: Junior college = 1, Bachelor’s degree = 2, Master’s degree = 3; Working units: CDC = 1, Hospital = 2, Primary health centers = 3, Others = 4; Working hours: ≤8 h = 0, >8 h = 1. 95% CI, Bootstrap confidence intervals with lower and upper limits. ^*^p < 0.05, ^**^p < 0.01, ^***^p < 0.001.

**Table 3:** Conditional indirect effects of the long working hours on depressive symptoms through job burnout at different level levels of social support.

Group	Effect	Boot SE	95% Boot CI
Low level of family support (M – 1SD)			
Low level of organizational support (*M* – 1 SD)	0.26	0.52	−0.75, 1.29
Mean level of organizational support (*M*)	0.67	0.44	−0.19, 1.52
High level of organizational support (M + 1 SD)	0.99	0.51	0.01, 2.00
Mean level of family support (*M*)			
Low level of organizational support (*M* – 1 SD)	0.14	0.38	−0.63, 0.89
Mean level of organizational support (*M*)	0.52	0.24	0.06, 0.99
High level of organizational support (*M* + 1 SD)	0.81	0.31	0.22, 1.44
High level of family support (M + 1 SD)			
Low level of organizational support (*M* – 1 SD)	0.03	0.46	−0.87, 0.91
Mean level of organizational support (*M*)	0.38	0.29	−0.17, 0.96
High level of organizational support (*M* + 1 SD)	0.66	0.28	0.12, 1.24

95% Boot CI, Bootstrap confidence intervals with lower and upper limits.

### 2.4. Common method bias

The psychological and social support data were collected simultaneously through self-rating questionnaire, thus common method bias (CMB) was a potential problem. In order to minimize this effect as much as possible, we controlled from the procedures, such as anonymous testing, careful questionnaire arrangement, etc. Meanwhile, Harman’s one-factor test was applied to test for the CMB (Podsakoff et al., 2003). The first factor explains variance less than 50%, suggesting that common method variance does not represent a problem in the study. Principal component analysis with varimax rotation results showed that the first factor accounted for 37.641% of the variance, indicating CMB was not significant in our study.

## 3. Results

### 3.1. Basic characteristics

A total of 992 respondents participated in the survey, mean age was 37.74 years and 68.85% were women and most were married (82.56%) (Table 4). Many participants had bachelor’s degree or below (99.4%) and most were hospitals staff (64.92%). Average working hours of the respondents was 9.46 h/day and 56.96% worked more than 8 h per day. 39.62% perceived high family support and moderate organizational support (mean score 4.22). Among the participants, 65.83% had burnout symptoms (MBI-GS mean score > 1.5) and 49.8% had depressive symptoms (PHQ-9 score > 5).

**Table 4:** Basic characteristics of frontline medical staff (*n* = 992).

Variables		*N* (%)	Depressive symptoms
Gender	Male	309 (31.15)	152 (49.19)
	Female	683 (68.85)	342 (50.07)
Age (years)	<30	228 (22.98)	111 (48.68)
	30–39	370 (37.30)	188 (50.81)
	40–49	246 (24.80)	131 (53.25)
	≥50	148 (14.92)	64 (43.24)
Marital status	Unmarried	154 (15.52)	74 (48.05)
	Married	819 (82.56)	411 (50.18)
	Others	19 (1.92)	9 (47.37)
Education	Junior college	496 (50.00)	228 (45.97)
	Bachelor’s degree	490 (49.40)	263 (53.67)
	Master’s degree	6 (0.60)	3 (50.00)
Working units	CDC	119 (12.00)	80 (67.23)
	Hospital	644 (64.92)	296 (45.96)
	Primary health centers	173 (17.44)	94 (54.34)
	Others	56 (5.65)	24 (42.86)
Hours of work per day	≤8 h	427 (43.04)	185 (43.33)
	>8 h	565 (56.96)	309 (54.69)
Family support	Very unsupported	19 (1.92)	9 (47.37)
	Non-support	36 (3.63)	27 (75.00)
	General	217 (21.88)	155 (71.43)
	Supported	327 (32.96)	177 (54.13)
	Very supported	393 (39.62)	126 (32.06)
Job burnout symptoms	Yes	653 (65.83)	416 (63.71)
	No	339 (34.17)	78 (23.01)
Total		992 (100)	494 (49.80)

CDC, centers for disease control and prevention.

### 3.2. Bivariate correlations predictor, mediator, moderator, and depressive symptoms

The correlations among work-related demands, social support and depressive symptoms are presented in Table 5. Long Working hours were positively correlated with job burnout (*r* = 0.093, *p* < 0.001) and depressive symptoms (*r* = 0.134, *p* < 0.001). Depressive symptoms was significantly and positively correlated with job burnout (*r* = 0.605, *p* < 0.001), and negatively correlated with family support (*r* = −0.446, *p* < 0.001) and organizational support (*r* = −0.408, *p* < 0.001).

**Table 5:** Bivariate correlations among predictor, mediator, moderator, and depressive symptoms.

Variables	Mean (SD)	1	2	3	4	5
1. Working hours	9.46 ± 3.09	1				
2. Job burnout	2.00 ± 1.10	0.093^**^	1			
3. Family support	4.05 ± 0.96	−0.092^**^	−0.446^***^	1		
4. Organizational support	4.22 ± 0.49	−0.037	−0.408^***^	0.300^***^	1	
5. Depressive symptoms	6.16 ± 6.59	0.134^***^	0.605^***^	−0.344^***^	−0.243^***^	1

*p < 0.05, **p < 0.01, ***p < 0.001.

### 3.3. Job burnout as mediator

The mediation regression results of were shown in Table 1. Long working hours was significantly and positively related to depressive symptoms (*β* = 1.83, 95% CI: [0.99 ~ 2.68]) after adjusting for covariates (see Model 1 in Table 1), providing support for Hypothesis 1. Working longer hours significantly predicted higher job burnout (*β* = 0.14, 95% CI [0.11, 0.40]) (see Model 2 in Table 1), which, in turn, was significantly associated with high PHQ-9 score (*β* = 3.92, 95% CI [3.64, 4.21]) (Model 3 in Table 1). When job burnout was added, the direct effect of long working hours on depressive symptoms was still significant. Moreover, the bias-corrected percentile Bootstrap test showed the mediating effect was significant (indirect effect = 1.00, 95% CI [0.47, 1.54]), which indicated that job burnout played a partial mediating role in this relationship. Thus, hypothesis 2 was supported.

### 3.4. Social support as moderator

Moderated mediation regression results were shown in Table 2 and Figure 2. As the moderator of social support variables and the interaction terms were entered into the models, long working hours still had significant direct effects on job burnout (*β* = 0.14, 95% CI [0.02, 0.26]) and depressive symptoms (*β* = 0.81, 95% CI [0.17, 1.45]) (see Model 4a and Model 4b in Table 2), but the two interactions of social support and long working hours (family support * long working hours, organizational support * long working hours) failed to predict job burnout and depressive symptoms, suggesting hypothesis 3a and hypothesis 3c were rejected. Both family support and organizational support were significantly related to lower job burnout (see Model 4a in Table 2 and Figure 2), indicating social support could reduce frontline medical staff from the negative effect of job-related burnout, the main effect of social support was verified.

Model 4b showed that depressive symptoms was not only significantly related to long working hours and job burnout, but also was linked to the interaction of job burnout and family support (*β* = −0.35, 95% CI [−0.64, −0.06]) and the interaction of job burnout and organizational support (*β* = −0.82, 95% CI [−1.37, −0.27]), which indicated that social support buffered the detrimental effect of job burnout on depressive symptoms, providing support to hypothesis 3b. Specifically, Figures 3, 4 showed the conditional direct effects of job burnout (M) on depressive symptoms (Y) at different moderator levels, where there were stronger direct effects in the lower level of social support than higher level social support. It can be inferred from the plots that, with higher social support and lower job burnout, there was attenuating effect on depressive symptoms. Therefore, in the presence of social support, the direct effects of job burnout on depressive symptoms were weaker.

The value of moderated mediating effects were calculated and results in Table 3 further verified that the indirect effects of the long working hours on depressive symptoms through job burnout were moderated by family support and organizational support. The indirect effect was significant for participants with high level family support and high level organizational support (estimated effect = 0 0.66, 95% CI [0.12, 1.24]), whereas this indirect effect was not significant for participants with low levels of family support and low level organizational support (estimated effect = 0.26, 95% CI [−0.75, 1.29]). Therefore, hypothesis 3 was supported. In addition, the bootstrap analysis revealed both the interaction of family support and job burnout (*β* = −0.02, 95% CI [−0.05, −0.01]) and interaction of family support and job burnout (*β* = −0.05, 95% CI [−0.08, −0.02]) had significant and negative indirect effect on depressive symptoms. These results highlighted that in the presence of high social support, the effects of long working hours on depressive symptoms through job burnout declined.

## 4. Discussion

To our knowledge, this is the first study to examine the mediating role of job burnout and the moderating role of social support (family support and organizational support) on the relationship between the long working hours and depressive symptoms among frontline medical staff during the COVID-19 pandemic in China. Based on the JD-R model and social support theory, our research hypothesized negative impact of job-related demands on depressive symptoms and employed moderated mediation analysis to test the model to reveal several findings. First, long working hours were significantly related to high level of depressive symptoms among frontline medical staff. Second, job burnout, as an independent mediator, significantly mediated the effect of long working hours on depression. Third, both family support and organizational support significantly moderated the detrimental effect of job burnout on depressive symptoms. Fourth, social support buffered the mediating pathways of long working hours on depressive symptoms through job burnout. In the presence of high social support, the harmful effects of long working hours on depressive symptoms declined through reducing burnout.

### 4.1. Long working hours and depressive symptoms

A meaningful findings of our study is that frontline medical staff worked longer hours with increased depressive symptoms. Previous limited studies have demonstrated work-related stressors and long working hours was associated with physicians’ psychological health (e.g., burnout and anxiety) (Kurzthaler et al., 2021; Ishikawa, 2022; Tsou, 2022). The COVID-19 pandemic led to extra hours on medical staff, which increased their mental health problems (Abbas et al., 2022). More work-related demands and lack of resource support may be the main reasons for depressive symptoms among frontline medical staff. In addition, the prevalence of depression among frontline medical staff was 49.8% in this study, which is higher than the prevalence of health care workers in Singapore (8.9%) and in the Indo-Pacific region (29.5–39.1%) (Tan et al., 2020; Kanneganti et al., 2022; Zou et al., 2022). Various measurements used in these studies may capture different aspects of depressive symptoms, which may explain the inconsistent results in some degree. During COVID-19 pandemic, overtime work and heavy workload have resulted in increased risk of many mental disorders in frontline medical staff. Thus, further investigating whether and how working hours impact on medical staff mental health well-being would improve our understanding of the risk of long working hours.

### 4.2. The mediating effects of job burnout

The main contribution of our study is that demonstrated robust mediating role of job burnout in the associations between long working hours and depressive symptoms, in both mediation analysis and moderated mediation analysis, which provide evidence for understanding the detrimental effect of long working hours on individuals’ mental health. Long working hours can lead to physical and mental exhaustion in the frontline medical staff, which can lead to a negative attitude toward work and the workplace. They may no longer devote themselves to their work with the same energy as in the past, which eventually leads to job burnout. A recent survey research from 481 Australian physicians indicated that a larger proportion of frontline general practitioners than specialists had high job burnout due to longer working hours during the COVID-19 pandemic (Ishikawa, 2022). When employees burned out about their work, they may report severe physical and psychological health problems. Some studies have demonstrated mild and moderate burnout was related to increased risk of anxiety and depression (Hu et al., 2011; Hyman et al., 2011; Brauchli et al., 2015). Based JD-R theory, burnout models and aforementioned studies (Hu et al., 2011; Brauchli et al., 2015), burnout may act as a mediating role in the health-impairment process. Our study have confirmed that burnout had a mediating role in the effect of long working hours on depressive symptoms with a large sample of 992 frontline medical staff in China. The findings help elucidate key underlying mechanisms and pathways linking job-related demands and depressive symptoms among frontline medical staff.

### 4.3. The moderating effect of social support

Another interesting contribution of our study is that we conducted a moderated mediation model to explore the buffering role of social support and interactions between job demands and social support in the relationship between long working hours and depressive symptoms, which broadened the JD-R model by investigating the combination effect of job related demand and work resource. Family and organizational support are both crucial work resources. The main effect model of social support suggested that increasing social support is beneficial to the development of positive psychological qualities and behaviors, regardless of whether the individual is in a negative situation or not (Gergen et al., 1998). Most studies have suggested that lack of organizational support generally led to less job burnout and lower depressive (Park et al., 2009; Giusti et al., 2020; Gu et al., 2020). The present study also found both family support and organizational support were associated with lower job burnout. In addition, our findings further examined that both family support and organizational support not only buffered the negative impact of job burnout on depressive symptoms, but also buffered the indirect effect of long working hours on depressive symptoms by reducing job burnout. High levels of family and organizational support may help medical staff feel accepted by others and the group, increase self-efficacy and job motivation, and not have a serious effect on mental health even when stressful occur (Zhang et al., 2020; Huang et al., 2022). Thus, in this study, the direct or indirect impact of long working hours and job burnout on depressive symptoms was highly associated and social support buffered these associations among frontline medical staff. This findings provide evidence that more assistance and support to frontline medical staff, as well as paying more attention to their family life situation and daily work could attenuate the negative effect of job-related demands and burnout on their physical and psychological health well-being.

## 5. Limitations

The present study has many limitations. First, we used a cross-sectional design to investigate the relationship between working hours, job burnout, family support, organizational support, and depression. Even though moderated mediation analysis can predict the relationship among the variables, the causality through cross-sectional studies could not be captured. Future studies should be invest more effort in experimental manipulating job characteristics using a longitudinal follow-up approach to verified whether and how job demands impact on health-impairment. Second, the generalizability of our findings is limited because we only performed this study in the northern Jiangsu Province. Future research should expand the scope and perform tracking studies. Third, the data were measured using self-report scales. Even though the results of Harman’s single-factor test showed no serious common method bias, future research should use objective measurements. Furthermore, the family support variable in this study was measured by a single item, and future studies could employ multiple entries to measure this variable to avoid bias. Despite the aforementioned limitations, our findings are reliable because the samples were representative. We selected a large cohort from different health care units, which included hospitals and CDC, and had a high response rate.

## 6. Implications

The findings of this study have important theoretical and practical implications for scientific prevention and intervention of mental health to frontline medical staff during the regular prevention and control phase of the COVID-19 pandemic. First, this study contributes to the literature by providing the high prevalence of job burnout and depression in the frontline medical staff, Chinese healthcare administrators should be more concerned about the deteriorating psychological well-being of frontline medical staff and assess their psychological well-being status regularly. Second, we found the significant effect of long working hours on depressive symptoms. Organizations should be aware of the potential negative effect of the additional working hours associated with the COVID-19 pandemic on the mental health of the frontline medical staff and arrange their working hours reasonably and effectively. Third, the mediating effect of job burnout on the association between working hours and depressive symptoms suggests that job burnout and long working hours must be considered concurrently to prevent depression. This mechanism provides a theoretical implication for why long working hours may lead to mental health disorders. Therefore, organizations should measure the three dimensions of job burnout in the frontline medical staff regularly. For individuals with a high level of burnout, extra training jobs, educational programs, or psychological counseling should be provided. Finally, our study confirmed the protective effects of family and organizational support between working hours, job burnout and depression, which provides a theoretical contribution to the social support theory. Family and medical units should provide family and organizational support to the medical staff at multiple levels, including organizational norms, instrumental support, and emotional support to alleviate job burnout levels and negative psychological emotions.

## 7. Conclusion

Working long hours among frontline medical physicians and primary health workers is very common phenomenon in China, especially during the COVID-19 pandemic. However, there is a growing discontent toward working long hours and it is becoming increasing challenging for individuals to maintain their physical and mental health. This study provides evidence for the hypothesis that job burnout mediated the detrimental effect of job-related demands (long working hours) on mental health of frontline medical staff and examined two sources of important resources (family support and organizational support) buffering this harmful effect on individuals’ depressive symptoms by reducing their job burnout. Our findings enrich JD-R model and social support theory literature by uncovering the underlying mechanisms and pathways how combinations of job burnout and social support (family support and organizational support) attenuated the deleterious effect of job-related demands on depressive symptoms. This findings can guide healthcare organizations in designing appropriate practices and working environments to enhance employees’ well-being, especially as frontline medical staff workload and work hours continue increase during the COVID-19 pandemic.

## Data availability statement

The raw data supporting the conclusions of this article will be made available by the authors, without undue reservation.

## Ethics statement

The studies involving human participants were reviewed and approved by the Ethics Committee of Nantong University. The patients/participants provided their written informed consent to participate in this study.

## Author contributions

YG and QM: conception and research design. CY and HJ: provision of study materials or patients. JJ and XC: collection and assembly of data. CY and JJ: data analysis and interpretation. CY, JJ, XC, HJ, QM, and YG: manuscript writing. All authors contributed to and have approved the final manuscript.

## Funding

This work was supported by the National Natural Science Foundation of China (grant no. 71603137) and the Social Science Foundation of Jiangsu Province (grant no. 19GLB026).

## Conflict of interest

The authors declare that the research was conducted in the absence of any commercial or financial relationships that could be construed as a potential conflict of interest.

## Publisher’s note

All claims expressed in this article are solely those of the authors and do not necessarily represent those of their affiliated organizations, or those of the publisher, the editors and the reviewers. Any product that may be evaluated in this article, or claim that may be made by its manufacturer, is not guaranteed or endorsed by the publisher.
